# Spatial–temporal pattern of cutaneous leishmaniasis in Brazil

**DOI:** 10.1186/s40249-021-00872-x

**Published:** 2021-06-16

**Authors:** Tatiana P. Portella, Roberto A. Kraenkel

**Affiliations:** 1 Departamento de Ecologia, Instituto de Biociências, Universidade de São Paulo, São Paulo, Brazil; 2 Instituto de Física Teórica, Universidade Estadual Paulista, São Paulo, Brazil

**Keywords:** Cutaneous leishmaniasis, Spatiotemporal cluster, Emerging hotspot, Temporal trend, Brazil

## Abstract

**Background:**

Cutaneous leishmaniasis (CL) is a vector-borne disease classified by the World Health Organization as one of the most neglected tropical diseases. Brazil has the highest incidence of CL in America and is one of the ten countries in the world with the highest number of cases. Understanding the spatiotemporal dynamics of CL is essential to provide guidelines for public health policies in Brazil. In the present study we used a spatial and temporal statistical approach to evaluate the dynamics of CL in Brazil.

**Methods:**

We used data of cutaneous leishmaniasis cases provided by the Ministry of Health of Brazil from 2001 to 2017. We calculated incidence rates and used the Mann–Kendall trend test to evaluate the temporal trend of CL in each municipality. In addition, we used Kuldorff scan method to identify spatiotemporal clusters and emerging hotspots test to evaluate hotspot areas and their temporal trends.

**Results:**

We found a general decrease in the number of CL cases in Brazil (from 15.3 to 8.4 cases per 100 000 habitants), although 3.2% of municipalities still have an increasing tendency of CL incidence and 72.5% showed no tendency at all. The scan analysis identified a primary cluster in northern and central regions and 21 secondary clusters located mainly in south and southeast regions. The emerging hotspots analysis detected a high spatial and temporal variability of hotspots inside the main cluster area, diminishing hotspots in eastern Amazon and permanent, emerging, and new hotspots in the states of Amapá and parts of Pará, Roraima, Acre and Mato Grosso. The central coast the state of Bahia is one of the most critical areas due to the detection of a cluster of the highest rank in a secondary cluster, and because it is the only area identified as an intensifying hotspot.

**Conclusions:**

Using a combination of statistical methods we were able to detect areas of higher incidence of CL and understand how it changed over time. We suggest that these areas, especially those identified as permanent, new, emerging and intensifying hotspots, should be targeted for future research, surveillance, and implementation of vector control measures.

**Graphic abstract:**

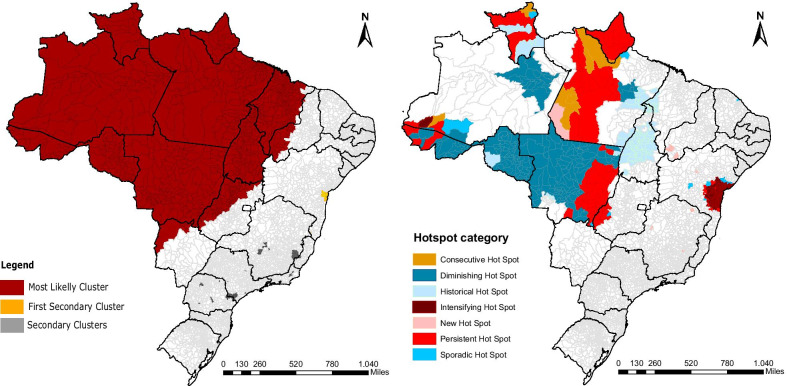

**Supplementary Information:**

The online version contains supplementary material available at 10.1186/s40249-021-00872-x.

## Background

Cutaneous leishmaniasis (CL) is a vector-borne disease infecting from 0.7 to 1.2 million people per year [1] and it is classified as one of the most neglected tropical diseases [2]. CL is caused by an obligate parasite of the genus *Leishmania* and is transmitted to humans by the bite of infected female sandflies [3]. It has three primary clinical forms: (1) localized cutaneous, which is characterized by one or multiple ulcerated skin lesions; (2) diffuse, in which the patients present nodular lesions disseminated all over the body; and (3) mucocutaneous, which is characterized by necrosis of the nasopharyngeal mucous tissue [4]. CL is treatable and has a low mortality rate, but it can generate social stigma and depressive disorder due to permanent scars and facial disfigurement [5].

CL occurs in at least 83 countries, mainly in the tropical, arid, and Mediterranean regions [6]. Recently, the World Health Organization classified this disease as a public health problem in the Americas and estimated that between 187 200 and 307 800 cases occur every year [7, 8]. Most of CL cases in the Americas occur in Brazil, with an annual mean of newly infected people of 28 166 from 2001 to 2005 and 21 632 from 2006 to 2011 [7]. Brazil is also one of the countries with the highest number of cases of CL in the world, and alongside Afghanistan, Algeria, Colombia, Iran, Syria, Ethiopia, Northern Sudan, Costa Rica, and Peru account together for 70–75% of the global incidence of the disease [1].

A number of epidemiologic studies report that spatio-temporal distribution of CL is an important factor to be considered when planning mitigation measures to control this disease in seriously affected countries. For example, it has been shown that despite the higher incidence of CL in Costa Rica, Colombia and Syria the majority of the cases are aggregated in spatio-temporal clusters restricted to a few regions scattered throughout these countries [9–11]. The only long-term study assessing spatial and temporal distribution of CL cases in Brazil analyzed temporal variation for the entire country and spatial distribution of cases aggregated by Brazilian states [7]. A few studies in Brazil assessed spatial and temporal variability of CL incidence in a higher resolution (i.e. for each municipality), and all of them were restricted to individual Brazilian states, namely: Paraná [12], Minas Gerais [13], Acre [14] and Amazonas [15].

According to the American Cutaneous Leishmaniasis Surveillance Program in Brazil [16], identifying territorial units of major epidemiological significance is essential to provide guidelines for public health policies as prevention, surveillance, and control measures. In order to contribute to this effort in this study we analyzed a dataset ranging from 2001 to 2017 to investigate for the first time the spatial and temporal dynamics of CL at the municipality level in Brazil. Using exploratory maps, built with Geographic Information System (GIS), and spatial and temporal statistical methods, we determined in which areas in Brazil CL incidence is higher and whether it increased, stabilized or decreased over time. In addition to an up-to-date discussion on spatiotemporal aspects of CL, we also suggest which areas in Brazil might be important for future research, monitoring and control measures.

## Methods

### Study area

Brazil has 5570 municipalities distributed in 26 states which are grouped in five macroregions: north, northeast, midwest, southeast and south. These macroregions were established in the 1970s by IBGE (*Instituto Brasileiro de Geografia e Estatística*) to facilitate the survey and dissemination of statistical data in Brazil to add a perspective to the understanding of national territory organization and to help the federal government, as well as states and municipalities, in the implementation and management of public policies and investments. In addition, Brazil has also an area called Legal Amazon, which was created by the government in the 1950s to prompt the development of socio-economic policies in this region. Currently, Brazilian Legal Amazon (BLA) area covers all states from North region (Amazonas, Acre, Rondônia, Roraima, Amapá, Tocantins and Pará), the state of Mato Grosso (Midwest) and part of Maranhão (Northeast). It covers 720 municipalities, with an approximate area of 5 217 423 km^2^, corresponding to 61% of the Brazilian territory [17].

### Data collection

A data set on individual-level, anonymized, cases of CL comprising a period ranging from 2001 to 2017 was provided by the Information System of Notifiable Disease (SINAN—*Sistema de Informação e Notificação de Agravos*) of the Ministry of Health of Brazil. Confirmed CL cases were grouped by year and municipality, and CL annual incidence was calculated with the estimated annual population data extracted from Brazilian Institute for Geography and Statistics (IBGE—*Instituto Brasileiro de Geografia e Estatística*).

Created in 1990, SINAN is the official nationwide platform for epidemiological surveillance of all compulsory notifiable diseases, which are defined by the National Compulsory Notification List of diseases. It is responsible for notification, investigation and, in the case of communicable diseases, follow-up and the outcome of patient's treatment. Notification is usually made by health professionals in the most peripheral administrative level (i.e. health care units) through standardized individual notification record by disease or condition type. All compulsory notifiable disease should be notified by the public, private and philanthropic health services. If the notifying health facility does not have computerized system, the physical records should be sent periodically to the municipal, regional or state office to be further digitalized and submitted to the national database [18].

Cutaneous leishmaniasis has been included by a specific federal statement on the national list of compulsory notifiable diseases since 2001 [19]. Only confirmed CL cases are notified, and they are diagnosed through clinical-epidemiological or laboratory criteria, mainly through direct parasitological examination, Montenegro Intradermal Test (MIT) or histopathological examination [20]. The dataset of confirmed CL cases is publicly available and can be accessed on the SINAN webpage (http://www2.datasus.gov.br/DATASUS/index.php?area=0203).

### Temporal trend analysis

First, we explored temporal trends of incidence for all country and for all municipalities affected by the disease. We used the LOESS curve fitting (locally weighted scatterplot smoothing) [21] to assess the tendency line of annual CL incidence in Brazil. To adjust the curve we used a combination of parameters to minimized an excessive weight in the smoothing and avoid over-adjustment. We found a better curve adjustment with an alpha value of 0.6. Since Brazil has thousands of municipalities, it is not feasible to visualize the time trend curves for each municipality individually. Therefore, we used the Mann Kendall Trend Test [22, 23] to evaluate if there was a significant temporal trend on the time series of each municipality, we categorized the municipalities according to their time trend and we plotted the obtained results on a map. The Mann–Kendall trend test is a non-parametric test for identifying trends in time series data and it works comparing the relative magnitudes of sample data rather than the data values themselves [24]. We used the statistical software R 3.6.3 (Lucent Technologies, Jasmine Mountain, USA) to perform the LOESS curve fitting and ARCGIS 10.4 (ESRI, Red-lands, CA, USA) to perform Mann Kendall Trend test.

### Spatial–temporal analysis

#### Scan statistic method

The spatial–temporal scanning method proposed by Kulldorffs [25] was used to find spatiotemporal clusters of CL by detecting an excess of cases in a given region and period of time. This method makes the assumption that the disease cases are generated by a non-homogenous Poisson process and, under the null hypothesis, the expected cases for each municipality are proportional to their population size. This method was implemented through a two-dimensional cylindrical window: an ellipse base, representing the potential cluster’s geographic areas and the height representing the period of study (number of years). The radius of the cylinder window varied in both spatial size and temporal length until it reached a previously defined maximum area of 15% of the population size and 90% of the study period.

Within each cylinder, the actual and expected number of disease cases, along with a Poisson generalized likelihood ratio (GLR) is calculated. The *P*-value for detected cluster was assessed using Monte Carlos simulation, where the maximum likelihood from the actual data was compared to the maximum likelihood from each 999 random simulated data sets generated under the null hypothesis. The cylinder with the maximum likelihood and with more than its expected number of cases was designated the most likely cluster. The other cylinders for which the likelihood value was statistically significant were defined as secondary clusters, and were ranked according to their likelihood ratio test statistics [25, 26]. Scan statistics analysis were performed using SatScan software v9.6 (Boston, MA, USA) and the results were visualized using ArcGIS 10.4 (ESRI, Redlands, CA, USA).

#### Emerging hotspot analysis

Before running the temporal hotspot analysis we transformed our data into a 3D cube format with bins fixed in space (x, y) and time (z). The space value (x, y) was assigned as the coordinates of each municipality and the time value (z) was the incidence of CL in each municipality per year of study. Thereafter, to evaluate spatiotemporal hotspots of Cutaneous Leishmaniasis incidence in each municipality we applied the Emerging Hot Spot Analysis tool (ArcGIS 10.4) using a combination of Getis ord Gi^*^ statistic and Mann Kendall Trend test.

The Getis ord Gi^*^ statistic [27] was used to identify areas of aggregation of higher incidence of CL in Brazil. This method works by summing the incidence value of one municipality and its neighbors comparing proportionally to the sum of incidence of all municipalities. When the local sum is much different than the expected, and that difference is too large to be the result of random chance, a statistically significant *Z* score is the result. The Getis-Ord Gi^*^ statistic generates *Z* scores (standard deviations) and *P* values (statistical probabilities) for each bin. A *Z* score above 1.96 means that there is a statically significant hot spot at a significant level of *P* < 0.05 and the larger a bin’s *Z*-score the more intense the hotspot is. Due to the cube structure of the data, neighboring bins exist both in time and in space. The emerging hotspot package used an adapted formula of kernel density search radius to define the neighborhood size in space [28] and temporal neighbors were defined using one prior time-step interval.

The Mann–Kendall statistic [22, 23] was used to evaluate a statistically significant temporal trend across the time series of *Z*-scores resulting from Getis-Ord Gi statistic. In this test, the bin value of the first time period is compared to the bin value for the second. If the first is smaller than the second, the result is a +1, if it is larger the result is -1, and if the two values are tied, the result is zero, indicating no trend in the values over time. Each pair of time steps was compared over the 17-year series, generating the Mann–Kendall statistics with associated trend *Z*-score and *P*-value for each bin. Based on the variance for the values in the bin time series, the number of ties, and the number of time periods, the observed sum is compared to the expected sum (zero) to determine if the difference is statistically significant or not. A small *P*-value indicates the trend is statistically significant, and the sign associated with the *Z*-score determines if the series has a monotonic increase (positive *Z*-score) or decrease trend (negative *Z*-score). The hotspot and trend results from the Getis Ord Gi^*^ and Mann–Kendall statistic were used to categorize each municipality [29] (see Table 1 for definition of the hotspots categories).

**Table 1:** Category name and definition of statistically significant hotspots representing different temporal states (ESRI ArcGIS Pro [30])

Hot spot category name	Definition
Intensifying	A location that has been a statistically significant hot spot for more than 90% of temporal series, including the final time step (2017). In addition, the intensity of clustering of high counts in each time step is increasing
Persistent	A location that has been a statistically significant hot spot for more than 90% of the temporal series, with no discernible trend indicating an increase or decrease in the intensity of clustering over time
Historical	The most recent time period is not hot, but at least ninety percent of the time-step intervals have been statistically significant hot spots
Consecutive	A location with a single uninterrupted run of statistically significant hot spot bins in the final time-step intervals. The location has never been a statistically significant hot spot prior to the final hot spot run and less than ninety percent of all bins are statistically significant hot spots
Sporadic	A location that is an on-again then off-again hot spot. Less than 90% of time series have been statistically significant hot spot
New	A location that is a statistically significant hot spot only on the last time steps of the time series
Diminishing	A location that has been a statistically significant hot spot for more than 90% of the time series. In addition, the intensity of clustering of high incidence in each time step is decreasing, or the most recent year is not hot

## Results

### CL cases distribution and temporal trends

**Fig. 1:**
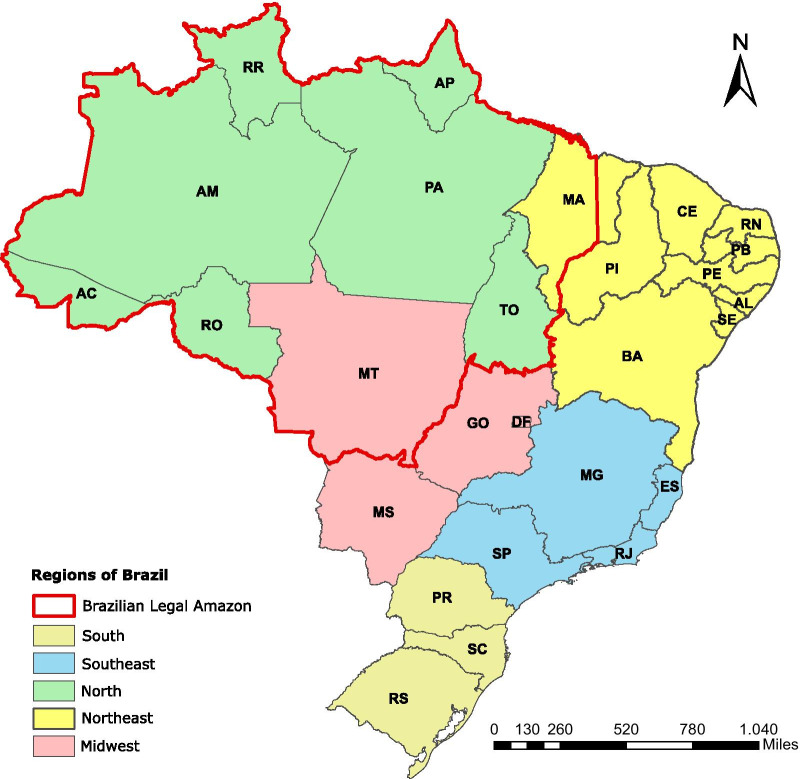
Map of Brazil and its territorial divisions. *AC* Acre, *AL*, Alagoas, *AP* Amapá, *AM* Amazonas, *BA* Bahia, *CE* Ceará, DF Distrito Federal, *ES* Espírito Santo, *GO* Goiás, *MA* Maranhão, *MT* Mato Grosso, *MS* Mato Grosso do Sul, *MG* Minas Gerais, *PA* Pará, *PB* Paraíba, *PR* Paraná, *PE* Pernambuco, *PI* Piauí, *RR* Roraima, *RP* Rondônia, *RJ* Rio de Janeiro, *RN* Rio Grande do Norte, *RS* Rio Grande do Sul, *SC* Santa Catarina, *SP* São Paulo, *SE* Sergipe, *TO* Tocantins

**Fig. 2:**
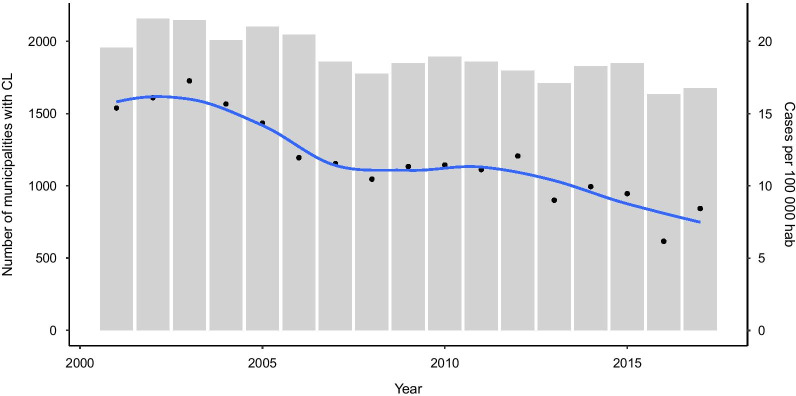
Number of municipalities with cutaneous leishmaniasis records and cutaneous leishmaniasis incidence rates per 100 000 habitants reported in Brazil during 2001–2017 period. The plotted points correspond to incidence rates and the lines consist in short term trends (fitted with locally estimated scatterplot smoothing LOESS).

During 2001–2017 period, a total of 379 571 cases of CL were registered in 73.8% of municipalities in Brazil (Fig. 1) with an average incidence of 11.86 cases per 100 000 inhabitants. The number of municipalities with confirmed CL cases was highest in 2002 (*n* = 2156) and lowest in 2016 (*n* = 1635). The CL incidence reported per year in Brazil showed that there is a tendency for reduction in number of cases. This curve also followed the fluctuation of the number of municipalities with CL-positive cases, with maximum value in the year 2003 and the minimum value in 2016 (Fig. 2).

**Fig. 3:**
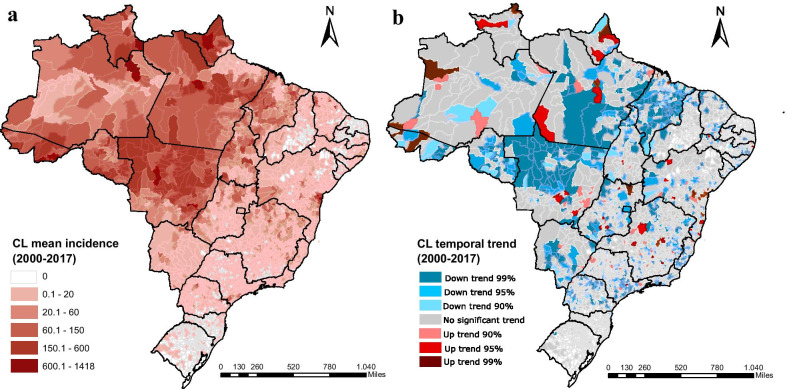
Spatial distribution of cutaneous leishmaniasis incidence mean rate per 100 000 inhabitants (**A**) and temporal trend of CL incidence stratified at the municipality level (**B**) during 2001–2017 period

Despite the wide distribution of cases approximately 80% of them occurred in only 10% of municipalities which were located mainly in Brazilian Legal Amazon (BLA), especially in the states namely Maranhão (MA), Mato Grosso (MT), Pará (PA) and Rondônia (RO), and outside of BLA in the state of Bahia (BA) (Fig. 3A). The temporal trend analysis for each municipality showed that 24.2% of them had a decrease in CL incidence, 3.2% had an increase, whereas 72.5% showed no tendency at all (Fig. 3B).

### Spatial–temporal clusters and hotspots

**Fig. 4:**
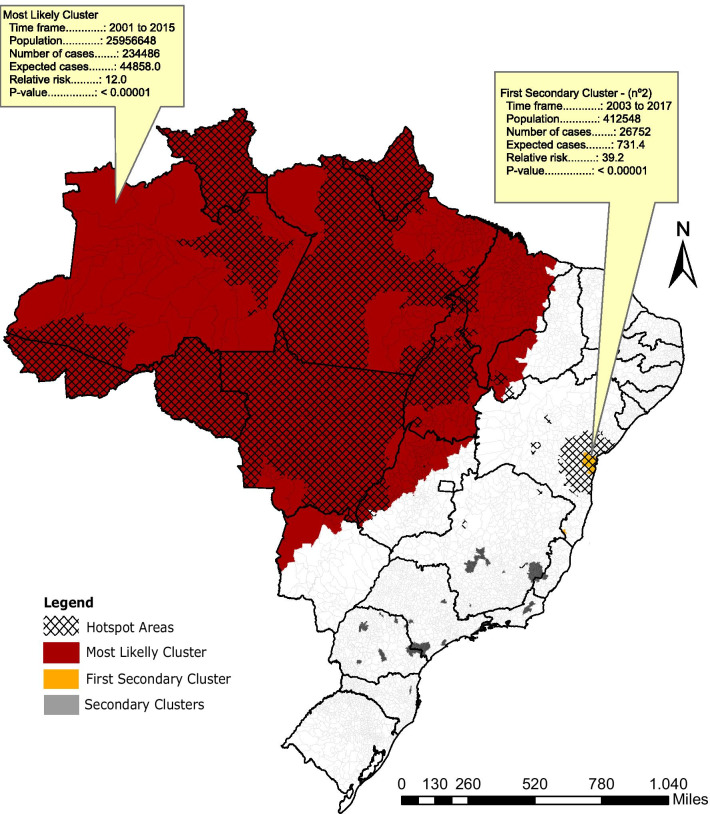
Cutaneous leishmaniasis space–time clusters (colored) obtained with spatial–temporal scan analysis, and hotspot areas (grid) obtained with emerging hotspot analysis (period of time ranging 2001–2017)

The most likely cluster detected has a relative risk of 12 (*P* < 0.001) for 2001–2015 period and covers the whole BLA (Fig. 4). In the secondary clusters the first cluster in the rank was located in the central coast of Bahia (BA) and has a relative risk of 39.2 (*P* < 0.001) for 2003–2017 period. The scan statistics also identified 20 more secondary clusters located in 90 municipalities scattered throughout mainly in the states of Minas Gerais (MG), São Paulo (SP), and Paraná (PR), with a relative risk ranging from 1.7 (from 2001 to 2004 and *P* < 0.05) to 22.7 (2001 to 2009 and *P* < 0.001) (Table 2) (Additional file 1).

**Table 2:** Cutaneous leishmaniasis secondary clusters detected in Brazil between 2001 and 2017 using the space–time scan statistic

Code	Center	State	Number of cities	Observed cases	Expected cases	Relative risk	Year	*P*-value
2	Nilo Peçanha	BA	20	26 752	731.42	39.2	2003–2017	< 0.001
3	Jussara	PR	9	1553	229.7	6.7	2001–2015	< 0.001
4	Itariri	SP	2	592	26.0	22.7	2001–2009	< 0.001
5	Conceição de Ipanema	MG	33	2828	933.2	3.0	2003–2017	< 0.001
6	Itaoca	SP	10	916	142.8	6.4	2002–2016	< 0.001
7	Cerro Azul	PR	1	324	30.3	10.6	2002–2016	< 0.001
8	Paraty	RJ	2	418	74.9	5.5	2001–2006	< 0.001
9	Rio Bonito do Iguaçu	PR	1	76	4.3	17.4	2004–2005	< 0.001
10	Prudentópolis	PR	1	97	10.8	8.9	2002–2003	< 0.001
11	Bandeirantes	PR	4	252	77.8	3.2	2001–2013	< 0.001
12	Sarutaiá	SP	3	75	10.7	6.9	2002–2003	< 0.001
13	Florestópolis	PR	2	57	6.3	8.9	2001–2002	< 0.001
14	Blumenau	SC	1	107	34.7	3.0	2006	< 0.001
15	Trajano de Moraes	RJ	4	72	15.6	4.6	2005–2006	< 0.001
16	Rio Acima	MG	1	58	12.9	4.4	2006–2017	< 0.001
17	Luz	MG	9	87	25.2	3.4	2014–2015	< 0.001
18	Conceição do Pará	MG	3	53	14.6	3.6	2002–2005	< 0.001
19	Pirassununga	SP	1	96	39.6	2.4	2001–2005	< 0.001
20	Careaçu	MG	1	17	1.3	12.3	2001–2002	< 0.001
21	Mangaratiba	RJ	2	130	73.1	1.7	2001–2004	< 0.05

*BA* Bahia, *CE MG* Minas Gerais, *PR* Paraná, *RJ* Rio de Janeiro, *SC* Santa Catarina, *SP* São Paulo

**Fig. 5:**
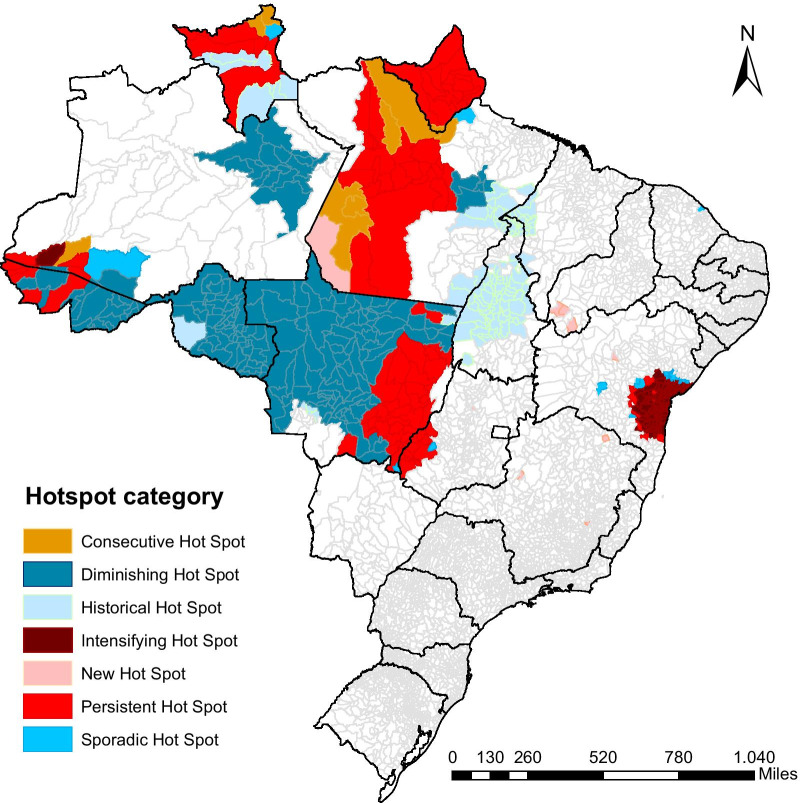
Cutaneous leishmaniasis hotspots using gets Ord Gi analysis and categorized according to their temporal trend using Mann Kendall Trend test (period of time ranging 2001–2017)

The emerging hotspot analysis identified significant areas of agglomeration of high incidence of CL in space and time (Fig. 5). These hotspots were located inside the most likely cluster area, excluding the central part of the Amazonas State (AM), east of Pará (PA), and most of Maranhão (MA) (Fig. 5). Another hotspot detected in the analysis was located in the central coast of Bahia, overlapping the area covered by the first in the rank of secondary clusters, as showed in Fig. 4.

A general decreasing trend in CL incidence was also found in the hotspot temporal analysis, whereas most of the hotspots identified were classified as historical or reducing hotspot. Most of the reducing hotspots were located in western BLA, although the temporal hotspot analysis also showed an intensifying hotspot placed in the coast of Bahia, in addition to persistent and recent hotspots located mainly in Amapá (AP), most of Roraima (RO), east of Acre (AC), west of Pará and south of Mato Grosso do Sul (MS) (Fig. 5).

## Discussion

To our knowledge, the present study describes for the first time spatial and temporal patterns of cutaneous leishmaniasis cases at municipality level for entirely Brazil. Despite Brazil being one of the countries with the highest incidence of CL in the world, most of the cases recorded from 2001 to 2017 were concentrated to specific regions. In addition, although we have found a general reduction of cases in Brazil, our results also showed that some municipalities had a stationary or an increasing tendency of CL incidence.

The spatiotemporal statistical scan showed that all municipalities in Brazilian Legal Amazon are included in the CL primary cluster. This is in agreement with a previous study that found that the states of Amapá, Roraima, Amazonas, Pará and Acre had the highest incidence of CL between 2001 and 2011 [7]. However, differently from this study, [7] evaluated the distribution of CL cases aggregated by Brazilian states and could not detect high incidence of CL in relatively small geographic areas in south, southeast and northeast of Brazil. Previous studies that accessed CL distribution in municipalities of Paraná [12] and Minas Gerais [13] also detected similar cluster areas as detected in this study, even using different methodologies. The only exception was the Jequitinhonha region and northern Minas Gerais, which were not included in any cluster in the present study, probably due to differences in the data sets composition. Therefore, the detection of similar regions with high incidence of CL in previous studies corroborates our results and highlights the relevance of the detected cluster areas to the surveillance and control of CL Leishmaniasis in Brazil.

Based on the emerging hotspot analysis, we found a high spatial and temporal heterogeneity of hotspots inside the primary cluster. This method did not find a large agglomeration of CL cases in the central part of Amazonas and eastern Pará, but it did find a predominance of reducing hotspot in western BLA and permanent and emerging hotspots in northern and eastern BLA. These results corroborate previous studies that showed a high spatial and temporal variability of CL cases in some states in northern Brazil. In accordance to our study, Teles et al*.* [15] found a smaller incidence of the disease in the interior of Amazonas and a high number of cases neighboring the State capital (Manaus), where we found a reducing hotspot. Additionally, similarly to this study Teles et al. [15] also found a higher number of CL cases in municipalities located in the southern border of Amazonas with Acre. Analyzing a period of time ranging from 2007 to 2013, Melchior et al. [14] found only one high-risk cluster of CL in southern part of Acre, but similarly to our study they also found that this region had a diminishing temporal trend of CL incidence.

Although a high incidence of CL has been historically found in the central coast of Bahia [31], this is the first time that this region has been identified as a cluster and an intensifying hotspot. It worth emphasizing that due to the risk of an increased trend of CL incidence and the risk of expansion of CL to neighboring regions, as previously reported for Rio de Janeiro state [32], the central cost of Bahia should be targeted as a high priority region for future surveillance and control measures of cutaneous leishmaniasis.

The clusters and hotspots found in this study may suggest that CL tend to be spatially and temporally distributed in Brazil and that environmental factors might play a role in modulating this distribution [33]. Previous studies have shown that the incidence of American cutaneous leishmaniasis is positively associated with the amount of forest cover, high diversity of possible reservoir mammal species and favorable climatic conditions for the development of sand-flies vectors, such as warm and hot weather and low annual seasonality of temperature and precipitation [34–36]. These environmental variables are typical of the Legal Amazon region in which we detected the most likely cluster of CL. Other studies have also shown positive association of CL cases and human environmental changes such as deforestation [37], settlements near forested areas [32, 38] and development of agriculture crops [39, 40]. These activities are predominant at the border of the Legal Amazon in a location called “Arc of Deforestation” [41], where we found most of the CL hotspots. Future studies should focus on understanding how these anthropogenic variables are affecting spatiotemporal dynamics of the disease in this area.

The presence of permanent and intensifying areas included in hotspots suggests that the strategies adopted for the control of CL in these regions might have not been efficient in reducing the number of cases. In Brazil, prevention strategies have mainly focused on diagnosis and treatment of the human disease, reducing morbidity, deformities and deaths, rather than on the control of vectors [42]. According to World Health Organization [43] an effective strategy for reducing human leishmaniasis is to control sand fly vectors and implementing health education in local communities, giving them important knowledge about individual protection measures and environmental modifications that can reduce the presence and frequency of vectors and hosts in the peridomicile and intradomicile areas [44]. Such an approach requires the proper knowledge of local epidemiology, which vector and host species are involved, their habitats, vector flight range, and seasonality [43]. However, the transmission patterns of CL are highly variable in Brazil, and little is known about the ecology of vector and mammalian reservoir species involved in the transmission cycle in many regions of the country [45, 46]. Therefore, the CL hotspot areas identified in this study should be considered as priorities in public health campaigns and should be used to guide future baseline studies about eco-epidemiology of CL transmission.

This study has two key limitations: First, is the use of secondary data, that can have different sources of errors including typing errors and the occurrence of underreporting cases due to the low coverage of public health agencies in remote areas of the country. However, the SINAN have seen considerable improvements in the last decades, including the mandatory notification of CL on both public and private service since 2001. Furthermore, CL diagnoses and treatment is free for all Brazilian citizens in the unified public health system (SUS), and thus the records of most of the CL cases in Brazil must have been included in SINAM [40]. Second, the analysis at the municipality level could not have revealed the actual site of disease focus. For more accurate analysis, future studies should examine the spatial distribution of CL at a finer scale (eg. zip code), however this data are poorly available in SINAN.

The use of two complementary approaches employed in this study allowed us to describe more precisely the spatial and temporal patterns of CL in Brazil. However, similar to all spatial clustering approaches, the statistical methods used in this study have limitations in accuracy and sensitivity. The scan is one of the most frequently employed methods of spatial and temporal cluster analysis and has the advantage of being adjusted for heterogeneous population and to look for clusters without specifying their specific localities overcoming the pre-selection bias [47]. However, this method has the disadvantage of not identify accurately the right format of very irregular clusters, and thus often report large clusters that contain several low-risk areas inside it [48, 49]. Therefore, we believe that this happened in our primary cluster due to the presence of some municipalities with low incidence rates. According to Han et al. [49], this issue could be improved by choosing ellipse radius values lower than 50%. However, due to the low population in northern Brazil (approximately 12.13% of Brazilian population) the analysis continued detecting a big and a few informative clusters, even when we reduce the radius length. In this sense, the gets Ord Gi analysis improved our study describing greater spatial and temporal heterogeneity in this area.

## Conclusions

In this study the spatial–temporal dynamics of CL in Brazil has been analyzed for at the municipality level and with a wider temporal range than previous works. The scan method showed that the main cluster is located in the Brazilian Legal Amazon and that most of the secondary clusters are located in the southern and southeastern regions of Brazil. The emerging hotspot analysis identified a higher spatiotemporal variability of hotspot inside the BLA. Using this method, it was also possible to detect diminishing hotspots in the east and persistent, emergent, and new hotspots, mainly in municipalities of the states of Pará, Rondônia, and Roraima, Acre and south of Mato Grosso. Furthermore, we showed that the central coast of Bahia was one of the most critical regions for CL due to the presence of a cluster with the highest rank in a secondary cluster and an intensifying hotspot. Despite a general decrease trend of CL cases in Brazil found in this study, the identification of areas of persistent, emergent, new, and intensifying hotspots suggest that the control measures in these regions are not effective in controlling the disease. The results reported herein may assist and guide future research about CL eco-epidemiology and the implementation of disease control measures in Brazil.

## Supplementary Information

**Additional file 1: S1. **Clusters detected from spatial-temporal scan analysis.

## Data Availability

All data used are publicly available. (http://www2.datasus.gov.br/DATASUS/index.php?area=0203).

## Abbreviations

BLA: Brazilian legal amazon
CL: Cutaneous leishmaniasis
IBGE: Brazilian Institute for Geography and Statistics (Instituto Brasileiro de Geografia e Estatística)
LOESS: Locally estimated scatterplot smoothing
SINAN: Information System of Notifiable Disease
AC: Acre
AL: Alagoas
AP: Amapá
AM: Amazonas
BA: Bahia
CE: Ceará
DF: Distrito Federal
ES: Espírito Santo
GO: Goiás
MA: Maranhão
MT: Mato Grosso
MS: Mato Grosso do Sul
MG: Minas Gerais
PA: Pará
PB: Paraíba
PR: Paraná
PE: Pernambuco
PI: Piauí
RR: Roraima
RP: Rondônia
RJ: Rio de Janeiro
RN: Rio Grande do Norte
RS: Rio Grande do Sul
SC: Santa Catarina
SP: São Paulo
SE: Sergipe
TO: Tocantins
