# Supplementary material for: Spatial–temporal pattern of cutaneous leishmaniasis in Brazil
Source: Infect Dis Poverty. 2021 Jun 16;10:86. doi: 10.1186/s40249-021-00872-x (PMC8207768; doi:10.1186/s40249-021-00872-x)
Supplement: Supplementary file 1 — Additional file 1: S1. Clusters detected from spatial-temporal scan analysis. [file 40249_2021_872_MOESM1_ESM.docx]

**Supplemental Material**

**S1. Clusters Detected from Spatial-temporal Scan analysis**

1.Location IDs included.: 130353, 130356, 130260, 130320, 130185, 130115, 130250, 130255,

130083, 130110, 130030, 130190, 130400, 130008, 130310, 130200,

130440, 130395, 130063, 130430, 130010, 130080, 130068, 130290,

130130, 140023, 140050, 140047, 140060, 130050, 130040, 130330,

150300, 130300, 130340, 150797, 130120, 150390, 130270, 140020,

150530, 140028, 150510, 140030, 150360, 140017, 150375, 130410,

150805, 130420, 140010, 150285, 130002, 150100, 150145, 130014,

140005, 130426, 130360, 150040, 150680, 140015, 150475, 150619,

130280, 140002, 140040, 150480, 130160, 150565, 130220, 130170,

130090, 150815, 150600, 140045, 140070, 130230, 130210, 150503,

130240, 150445, 130380, 130100, 150050, 510325, 150172, 110013,

160027, 160080, 110080, 110094, 110020, 150590, 130423, 110110,

150060, 510080, 510337, 130370, 150835, 110175, 510615, 510140,

110026, 130006, 510895, 110040, 510629, 150310, 110002, 510517,

160005, 110160, 160015, 110180, 150780, 110011, 510025, 110060,

110140, 510757, 150085, 160040, 510279, 110015, 130350, 110100,

110045, 160053, 110012, 130390, 160060, 110155, 160030, 150580,

510285, 110143, 150730, 110070, 510626, 110120, 160023, 150450,

130195, 110025, 110130, 110170, 510515, 510410, 510621, 510880,

110004, 110009, 150180, 150548, 510510, 110034, 110090, 510560,

510642, 160025, 160021, 110018, 510805, 510627, 160070, 510794,

150030, 150110, 110028, 510680, 110050, 110147, 510320, 110033,

110014, 130070, 160055, 110148, 110029, 110032, 150808, 510619,

160050, 160010, 110001, 150543, 150070, 160020, 130406, 150520,

510455, 150250, 110150, 150280, 110010, 110037, 110145, 130060,

150506, 510190, 150120, 120001, 130020, 150810, 150770, 150210,

150460, 110092, 150178, 510558, 110149, 150400, 120080, 150034,

110030, 510454, 510305, 150490, 510790, 130140, 120038, 150553,

150276, 510452, 150640, 110007, 510724, 150125, 510787, 510830,

150330, 150309, 150215, 110006, 150795, 120013, 150200, 150370,

120040, 120050, 510774, 150010, 150380, 150570, 150277, 110005,

120045, 110008, 510800, 150470, 150840, 150616, 510850, 150497,

510792, 510890, 130150, 510735, 150420, 150130, 150295, 150775,

120017, 110003, 120034, 110146, 510370, 150140, 150630, 150790,

510330, 150613, 150080, 150304, 150442, 150750, 510268, 510525,

150260, 150150, 150157, 150635, 150715, 510263, 150020, 510730,

150650, 150800, 150700, 150820, 510624, 510860, 170740, 211153,

150563, 150013, 150190, 171630, 120030, 150710, 510335, 150275,

120070, 150670, 150340, 150240, 150746, 150796, 150175, 510677,

510269, 150290, 170380, 172030, 170215, 150720, 150740, 150745,

150618, 150549, 172210, 211285, 170230, 150658, 120060, 150440,

150293, 170390, 171886, 150270, 150320, 170389, 170220, 510618,

510622, 150095, 170600, 150405, 170190, 150660, 150410, 171395,

120010, 120025, 510336, 150345, 171180, 150760, 170320, 150350,

170130, 171880, 170255, 150430, 210325, 171720, 150812, 170388,

150500, 171830, 171855, 150690, 510885, 130180, 510776, 150550,

170100, 510777, 150560, 210542, 150160, 150620, 172010, 170290,

170382, 170305, 510130, 510726, 120043, 510600, 510795, 171245,

210005, 150220, 171488, 510350, 171665, 172080, 170210, 150747,

150540, 170105, 172020, 150610, 510035, 171250, 150230, 150307,

510550, 510050, 170830, 172208, 211085, 210530, 170550, 510345,

510623, 150495, 120005, 150611, 210550, 210375, 171070, 171110,

171670, 171430, 150555, 171280, 510530, 172000, 170720, 170650,

150655, 510715, 150803, 510706, 170360, 211176, 510385, 210455,

510675, 510590, 210955, 171840, 510775, 510185, 510788, 170025,

150170, 510835, 510770, 171380, 170710, 170300, 172120, 210255,

210900, 170030, 510170, 510720, 510500, 210405, 170930, 210203,

510785, 171570, 510523, 150090, 510380, 170825, 150195, 210235,

172130, 210700, 171090, 510125, 510450, 170460, 170307, 510685,

171190, 170770, 171370, 171870, 210280, 210598, 510718, 510010,

510645, 510490, 171330, 510631, 211105, 171750, 172125, 130165,

510710, 210197, 170310, 171650, 170330, 210060, 510343, 510630,

210232, 510562, 171610, 171050, 170610, 120020, 510395, 211102,

171320, 211180, 120042, 172110, 510620, 510270, 210317, 150830,

170900, 510682, 210565, 171845, 171888, 171200, 120032, 210315,

171500, 210055, 120033, 210290, 210632, 510300, 171550, 510340,

170755, 210467, 510840, 171875, 210637, 120039, 210620, 171889,

210923, 170730, 510610, 210407, 510250, 211003, 210430, 172100,

210047, 510779, 210095, 211157, 210260, 170820, 170625, 210735,

510267, 510020, 510260, 210480, 510780, 210087, 210950, 211400,

170110, 171850, 210465, 170384, 170035, 170410, 211000, 510617,

211227, 171820, 210200, 210409, 120035, 170370, 210535, 171884,

210215, 170950, 510650, 211240, 510628, 170386, 210927, 210725,

171510, 510704, 510360, 210410, 210825, 210040, 211245, 210980,

210990, 171900, 510160, 210850, 210547, 171360, 170980, 510625,

210635, 510480, 210177, 170765, 210690, 172085, 510740, 510700,

210130, 210830, 210515, 211178, 211172, 170200, 210596, 211300,

510520, 210083, 210860, 522020, 172065, 210870, 210810, 171195,

210650, 210140, 210740, 210745, 210250, 211280, 170070, 210570,

210760, 210370, 210207, 210160, 171890, 211170, 210840, 210580,

210312, 211050, 171660, 211290, 171790, 210355, 210680, 210190,

211100, 521405, 210594, 211163, 510310, 210135, 211167, 210590,

210890, 210100, 171240, 172097, 210240, 521525, 211160, 210520,

210120, 510760, 210490, 172049, 171700, 210905, 520357, 210070,

510810, 521483, 210193, 510390, 210310, 210675, 210970, 211140,

510729, 210663, 170510, 210400, 211223, 171150, 210560, 510637,

210020, 510420, 210820, 172015, 171420, 211200, 210408, 211150,

520250, 210610, 211230, 210270, 210043, 210540, 210125, 211125,

211020, 211130, 210600, 521800, 210050, 220890, 220920, 210960,

210975, 521400, 210845, 210910, 210380, 510180, 522157, 510665,

521410, 210880, 520170, 220115, 520380, 211030, 210275, 210750,

172025, 210945, 510100, 211120, 210920, 210110, 210510, 210237,

170040, 210710, 171575, 520082, 510820, 521377, 520310, 520495,

210440, 211080, 210450, 210360, 211070, 210470, 520640, 171270,

521960, 210930, 520215, 522145, 211065, 520810, 171620, 210460,

171800, 520750, 521970, 171865, 521280, 210720, 211270, 520055,

520340, 510719, 521487, 211210, 210670, 210545, 210350, 510040,

510460, 211174, 210420, 521295, 170560, 210180, 210330, 210462,

221120, 170700, 520945, 521925, 510670, 510120, 520470, 500793,

520465, 521370, 211190, 521690, 521470, 521308, 211107, 520980,

172093, 520753, 211040, 210173, 220130, 521720, 210500, 522160,

520725, 220855, 521220, 210800, 171525, 210940, 521385, 220660,

220440, 210230, 521090, 210770, 521890, 210730, 220080, 520235,

211260, 520710, 521100, 522015, 521015, 510030, 220975, 521940,

210030, 210320, 210790, 521486, 522028, 211027, 521200, 521520,

210010, 520500, 520929, 171780, 521120, 520540, 221063, 521860,

210300, 520760, 220600, 220560, 211110, 521020, 521030, 210592,

500640, 522170, 521935, 521460, 521565, 210640, 521810, 210080,

520552, 220170, 170240, 520440, 521870, 521945, 211195, 520320,

520890, 520960, 220290, 172090, 520393, 520090, 220323, 220885,

520570, 220450, 220740, 500320, 220870, 500520, 220300, 210340,

521390, 510060, 220190, 521900, 210660, 520860, 220310, 210390,

210220, 521060, 210170, 520530, 220590, 520490, 521340, 521310,

521160, 170555, 220225, 220620, 171215, 521350, 220530, 500330,

170270, 171515, 210780, 521040, 220930, 522108, 210210

Coordinates...........: (-2.02981,-60.0234)

Semiminor axis........: 17.35

Semimajor axis........: 17.35

Angle (degrees).......: 0

Shape.................: 1.00

Time frame............: 2001/1/1 to 2015/12/31

Population............: 25956648

Number of cases.......: 234486

Expected cases........: 44858.05

Annual cases / 100000.: 61.2

Observed / expected...: 5.23

Relative risk.........: 12.06

Log likelihood ratio..: 266530.419225

Test statistic........: 266530.419225

P-value...............: < 0.00000000000000001

2.Location IDs included.: 292260, 293120, 290540, 291730, 291345, 293290, 292467, 293160,

293350, 292575, 291120, 290580, 292275, 291570, 292240, 291820,

291890, 290195, 293210, 292070

Coordinates...........: (-13.604,-39.1091)

Semiminor axis........: 0.50

Semimajor axis........: 0.76

Angle (degrees).......: 90.00

Shape.................: 1.50

Time frame............: 2003/1/1 to 2017/12/31

Population............: 412548

Number of cases.......: 26752

Expected cases........: 731.42

Annual cases / 100000.: 428.4

Observed / expected...: 36.58

Relative risk.........: 39.27

Log likelihood ratio..: 71184.659077

Test statistic........: 69746.436901

P-value...............: < 0.00000000000000001

3.Location IDs included.: 411300, 410550, 412720, 412610, 411240, 411160, 412530, 410730,

410750

Coordinates...........: (-23.6219,-52.4693)

Semiminor axis........: 0.27

Semimajor axis........: 0.27

Angle (degrees).......: 0

Shape.................: 1.00

Time frame............: 2001/1/1 to 2015/12/31

Population............: 132738

Number of cases.......: 1553

Expected cases........: 229.79

Annual cases / 100000.: 79.2

Observed / expected...: 6.76

Relative risk.........: 6.78

Log likelihood ratio..: 1646.521990

Test statistic........: 1646.521990

P-value...............: < 0.00000000000000001

4.Location IDs included.: 352330, 353720

Coordinates...........: (-24.2834,-47.1736)

Semiminor axis........: 0.062

Semimajor axis........: 0.062

Angle (degrees).......: 0

Shape.................: 1.00

Time frame............: 2001/1/1 to 2009/12/31

Population............: 25828

Number of cases.......: 592

Expected cases........: 26.08

Annual cases / 100000.: 265.9

Observed / expected...: 22.70

Relative risk.........: 22.73

Log likelihood ratio..: 1282.919384

Test statistic........: 1282.919384

P-value...............: < 0.00000000000000001

5.Location IDs included.: 311740, 316360, 311600, 313120, 316805, 313770, 314400, 315890,

312352, 315190, 320245, 314053, 316760, 320265, 315015, 315415,

313055, 320115, 320300, 315935, 315725, 311340, 313940, 313950,

317005, 310220, 316447, 316095, 310205, 315350, 313090, 320010,

317115

Coordinates...........: (-19.9326,-41.6908)

Semiminor axis........: 0.59

Semimajor axis........: 0.59

Angle (degrees).......: 0

Shape.................: 1.00

Time frame............: 2003/1/1 to 2017/12/31

Population............: 527176

Number of cases.......: 2828

Expected cases........: 933.26

Annual cases / 100000.: 35.5

Observed / expected...: 3.03

Relative risk.........: 3.05

Log likelihood ratio..: 1245.253649

Test statistic........: 1245.253649

P-value...............: < 0.00000000000000001

6.Location IDs included.: 352215, 410020, 354280, 352120, 350270, 352265, 350535, 350540,

351480, 412863

Coordinates...........: (-24.6393,-48.8413)

Semiminor axis........: 0.25

Semimajor axis........: 0.76

Angle (degrees).......: 80.00

Shape.................: 3.00

Time frame............: 2002/1/1 to 2016/12/31

Population............: 81355

Number of cases.......: 916

Expected cases........: 142.87

Annual cases / 100000.: 75.1

Observed / expected...: 6.41

Relative risk.........: 6.42

Log likelihood ratio..: 929.634175

Test statistic........: 805.086812

P-value...............: < 0.00000000000000001

7.Location IDs included.: 410520

Coordinates...........: (-26.0891,-52.8691)

Semiminor axis........: 0

Semimajor axis........: 0

Angle (degrees).......: 0

Shape.................: 1.00

Time frame............: 2002/1/1 to 2016/12/31

Population............: 17256

Number of cases.......: 324

Expected cases........: 30.35

Annual cases / 100000.: 125.0

Observed / expected...: 10.68

Relative risk.........: 10.68

Log likelihood ratio..: 473.697847

Test statistic........: 473.697847

P-value...............: < 0.00000000000000001

8.Location IDs included.: 330380, 355540

Coordinates...........: (-23.2221,-44.7175)

Semiminor axis........: 0.28

Semimajor axis........: 0.56

Angle (degrees).......: 30.00

Shape.................: 2.00

Time frame............: 2001/1/1 to 2006/12/31

Population............: 115803

Number of cases.......: 418

Expected cases........: 74.98

Annual cases / 100000.: 65.3

Observed / expected...: 5.57

Relative risk.........: 5.58

Log likelihood ratio..: 375.363460

Test statistic........: 353.896064

P-value...............: < 0.00000000000000001

9.Location IDs included.: 412215

Coordinates...........: (-25.4874,-52.5292)

Semiminor axis........: 0

Semimajor axis........: 0

Angle (degrees).......: 0

Shape.................: 1.00

Time frame............: 2004/1/1 to 2005/12/31

Population............: 15098

Number of cases.......: 76

Expected cases........: 4.35

Annual cases / 100000.: 204.8

Observed / expected...: 17.48

Relative risk.........: 17.48

Log likelihood ratio..: 145.799657

Test statistic........: 145.799657

P-value...............: < 0.00000000000000001

10.Location IDs included.: 412060

Coordinates...........: (-25.2111,-50.9754)

Semiminor axis........: 0

Semimajor axis........: 0

Angle (degrees).......: 0

Shape.................: 1.00

Time frame............: 2002/1/1 to 2003/12/31

Population............: 48906

Number of cases.......: 97

Expected cases........: 10.81

Annual cases / 100000.: 105.1

Observed / expected...: 8.98

Relative risk.........: 8.98

Log likelihood ratio..: 126.682508

Test statistic........: 126.682508

P-value...............: < 0.00000000000000001

11.Location IDs included.: 410240, 411100, 412310, 410010

Coordinates...........: (-23.1078,-50.3704)

Semiminor axis........: 0.14

Semimajor axis........: 0.21

Angle (degrees).......: 0

Shape.................: 1.50

Time frame............: 2001/1/1 to 2013/12/31

Population............: 51099

Number of cases.......: 252

Expected cases........: 77.89

Annual cases / 100000.: 37.9

Observed / expected...: 3.24

Relative risk.........: 3.24

Log likelihood ratio..: 121.820496

Test statistic........: 119.359222

P-value...............: < 0.00000000000000001

12.Location IDs included.: 355120, 353880, 410470

Coordinates...........: (-23.2721,-49.4763)

Semiminor axis........: 0.074

Semimajor axis........: 0.29

Angle (degrees).......: 60.00

Shape.................: 4.00

Time frame............: 2002/1/1 to 2003/12/31

Population............: 46644

Number of cases.......: 75

Expected cases........: 10.74

Annual cases / 100000.: 81.8

Observed / expected...: 6.98

Relative risk.........: 6.99

Log likelihood ratio..: 81.521507

Test statistic........: 65.217206

P-value...............: < 0.00000000000000001

13.Location IDs included.: 410800, 410280

Coordinates...........: (-22.8623,-51.3882)

Semiminor axis........: 0.080

Semimajor axis........: 0.24

Angle (degrees).......: -60.00

Shape.................: 3.00

Time frame............: 2001/1/1 to 2002/12/31

Population............: 26916

Number of cases.......: 57

Expected cases........: 6.38

Annual cases / 100000.: 104.7

Observed / expected...: 8.94

Relative risk.........: 8.94

Log likelihood ratio..: 74.235028

Test statistic........: 64.289420

P-value...............: < 0.00000000000000001

14.Location IDs included.: 420240

Coordinates...........: (-26.9155,-49.0709)

Semiminor axis........: 0

Semimajor axis........: 0

Angle (degrees).......: 0

Shape.................: 1.00

Time frame............: 2006/1/1 to 2006/12/31

Population............: 306872

Number of cases.......: 107

Expected cases........: 34.79

Annual cases / 100000.: 36.0

Observed / expected...: 3.08

Relative risk.........: 3.08

Log likelihood ratio..: 48.017940

Test statistic........: 48.017940

P-value...............: 0.000000000000089

15.Location IDs included.: 330590, 330460, 330530, 330480

Coordinates...........: (-22.0638,-42.0643)

Semiminor axis........: 0.15

Semimajor axis........: 0.58

Angle (degrees).......: 45.00

Shape.................: 4.00

Time frame............: 2005/1/1 to 2006/12/31

Population............: 67095

Number of cases.......: 72

Expected cases........: 15.65

Annual cases / 100000.: 53.9

Observed / expected...: 4.60

Relative risk.........: 4.60

Log likelihood ratio..: 53.534909

Test statistic........: 42.827927

P-value...............: 0.0000000000083

16.Location IDs included.: 315480

Coordinates...........: (-20.0876,-43.7878)

Semiminor axis........: 0

Semimajor axis........: 0

Angle (degrees).......: 0

Shape.................: 1.00

Time frame............: 2006/1/1 to 2017/12/31

Population............: 8834

Number of cases.......: 58

Expected cases........: 12.98

Annual cases / 100000.: 52.3

Observed / expected...: 4.47

Relative risk.........: 4.47

Log likelihood ratio..: 41.807699

Test statistic........: 41.807699

P-value...............: 0.000000000020

17.Location IDs included.: 313880, 310510, 312320, 314050, 312340, 311980, 313030, 316820,

310020

Coordinates...........: (-19.7911,-45.6794)

Semiminor axis........: 0.23

Semimajor axis........: 1.13

Angle (degrees).......: 36.00

Shape.................: 5.00

Time frame............: 2014/1/1 to 2015/12/31

Population............: 105020

Number of cases.......: 87

Expected cases........: 25.26

Annual cases / 100000.: 40.3

Observed / expected...: 3.44

Relative risk.........: 3.45

Log likelihood ratio..: 45.863223

Test statistic........: 34.184428

P-value...............: 0.000000016

18.Location IDs included.: 311760, 315140, 314580

Coordinates...........: (-19.7456,-44.8945)

Semiminor axis........: 0.091

Semimajor axis........: 0.091

Angle (degrees).......: 0

Shape.................: 1.00

Time frame............: 2002/1/1 to 2005/12/31

Population............: 33449

Number of cases.......: 53

Expected cases........: 14.65

Annual cases / 100000.: 42.4

Observed / expected...: 3.62

Relative risk.........: 3.62

Log likelihood ratio..: 29.797115

Test statistic........: 29.797115

P-value...............: 0.00000075

19.Location IDs included.: 353930

Coordinates...........: (-21.996,-47.4257)

Semiminor axis........: 0

Semimajor axis........: 0

Angle (degrees).......: 0

Shape.................: 1.00

Time frame............: 2001/1/1 to 2005/12/31

Population............: 70832

Number of cases.......: 96

Expected cases........: 39.69

Annual cases / 100000.: 28.3

Observed / expected...: 2.42

Relative risk.........: 2.42

Log likelihood ratio..: 28.486883

Test statistic........: 28.486883

P-value...............: 0.0000023

20.Location IDs included.: 311360

Coordinates...........: (-22.0424,-45.696)

Semiminor axis........: 0

Semimajor axis........: 0

Angle (degrees).......: 0

Shape.................: 1.00

Time frame............: 2001/1/1 to 2002/12/31

Population............: 6271

Number of cases.......: 17

Expected cases........: 1.37

Annual cases / 100000.: 145.1

Observed / expected...: 12.39

Relative risk.........: 12.39

Log likelihood ratio..: 27.157414

Test statistic........: 27.157414

P-value...............: 0.0000075

21.Location IDs included.: 330260, 330010

Coordinates...........: (-22.9594,-44.0409)

Semiminor axis........: 0.19

Semimajor axis........: 0.29

Angle (degrees).......: 90.00

Shape.................: 1.50

Time frame............: 2001/1/1 to 2004/12/31

Population............: 195255

Number of cases.......: 130

Expected cases........: 73.10

Annual cases / 100000.: 20.8

Observed / expected...: 1.78

Relative risk.........: 1.78

Log likelihood ratio..: 17.949910

Test statistic........: 17.587248

P-value...............: 0.033
